# Intergranular Strain Evolution During Biaxial Loading: A Multiscale FE-FFT Approach

**DOI:** 10.1007/s11837-017-2299-5

**Published:** 2017-03-10

**Authors:** M. V. Upadhyay, J. Capek, S. Van Petegem, R. A. Lebensohn, H. Van Swygenhoven

**Affiliations:** 1 Swiss Light Source, Paul Scherrer Institute, 5232 Villigen PSI, Switzerland; 2 Department of Physics of Materials, Charles University, 12116 Prague, Czech Republic; 3 Department of Neutron Physics, Nuclear Physics Institute ASCR, 25068 Řež, Czech Republic; 4 Materials Science and Technology Division, Los Alamos National Laboratory, Los Alamos, NM 87544 USA; 5 Neutrons and X-rays for Mechanics of Materials, IMX, Ecole Polytechnique Federale de Lausanne, 1012 Lausanne, Switzerland

## Abstract

Predicting the macroscopic and microscopic mechanical response of metals and alloys subjected to complex loading conditions necessarily requires a synergistic combination of multiscale material models and characterization techniques. This article focuses on the use of a multiscale approach to study the difference between intergranular lattice strain evolution for various grain families measured during in situ neutron diffraction on dog bone and cruciform 316L samples. At the macroscale, finite element simulations capture the complex coupling between applied forces and gauge stresses in cruciform geometries. The predicted gauge stresses are used as macroscopic boundary conditions to drive a mesoscale full-field elasto-viscoplastic fast Fourier transform crystal plasticity model. The results highlight the role of grain neighborhood on the intergranular strain evolution under uniaxial and equibiaxial loading.

## Introduction

Metals and alloys used for engineering purposes are often subjected to biaxial stress states and strain path changes during their fabrication or under service conditions. These complex strain paths, coupled with elastic/plastic anisotropy of polycrystals, result in heterogeneous distributions of intergranular strains, governing macroscopic response such as yield strength, work hardening, etc. Although biaxial testing is increasingly used to study macroscopic behavior of materials,^1^–^3^ limited research efforts have been directed toward understanding the underlying microstructure and intergranular strain evolution.^4^–^6^

In-situ neutron and synchrotron x-ray diffraction are well established techniques to study internal stress and microstructure evolution.^7^–^9^ The evolution of diffraction peak positions, width, and intensity can provide insight into the average intergranular and intragranular strains and texture evolution within different grain families;^10^ these grain families are classified according to their crystallographic orientation with respect to the diffraction vector. In the present work, the focus is on intergranular strains, also known as lattice or micro-strains, in differently oriented grain families. The average lattice strain of a grain family represents the fraction of applied load, i.e., type-I or macroscopic stresses, shared by that grain family. Elastic anisotropy, plastic slip, grain neighborhood interactions and the direction of loading significantly influence this evolution. Average lattice strain evolution during uniaxial loading has been studied for a variety of materials.^7^–^12^

Recently, a biaxial testing rig was developed to deform cruciform samples during in-situ neutron diffraction measurements.^13^ Cruciform samples of 316L austenitic stainless steel were deformed under uniaxial and biaxial monotonic tensile loading and strain path changes.^5^ The results showed that lattice strain evolution under monotonic equibiaxial tension (EQUI) is significantly different from uniaxial tension in a dog-bone (DB) sample.

A comprehensive understanding of the relationship between the biaxial stress ratio and lattice strain evolution can be achieved by combining in-situ diffraction studies with crystal plasticity modeling. Mesoscale models such as the mean-field elasto-plastic self-consistent model,^14^^,^ 15 the mean-field elasto-viscoplastic self-consistent model,^16^ the full-field crystal plasticity finite element (FE) model,^17^ the full-field elasto-viscoplastic fast Fourier transform (EVP-FFT) model,^18^ etc., have been used to understand the lattice strain evolution during uniaxial loading for different material systems. In this work, the computationally efficient full-field EVP-FFT model is used.^19^ The EVP-FFT is designed to study representative volume elements (RVEs) of polycrystals subjected to strain rate or stress boundary conditions.

**Fig. 1:**
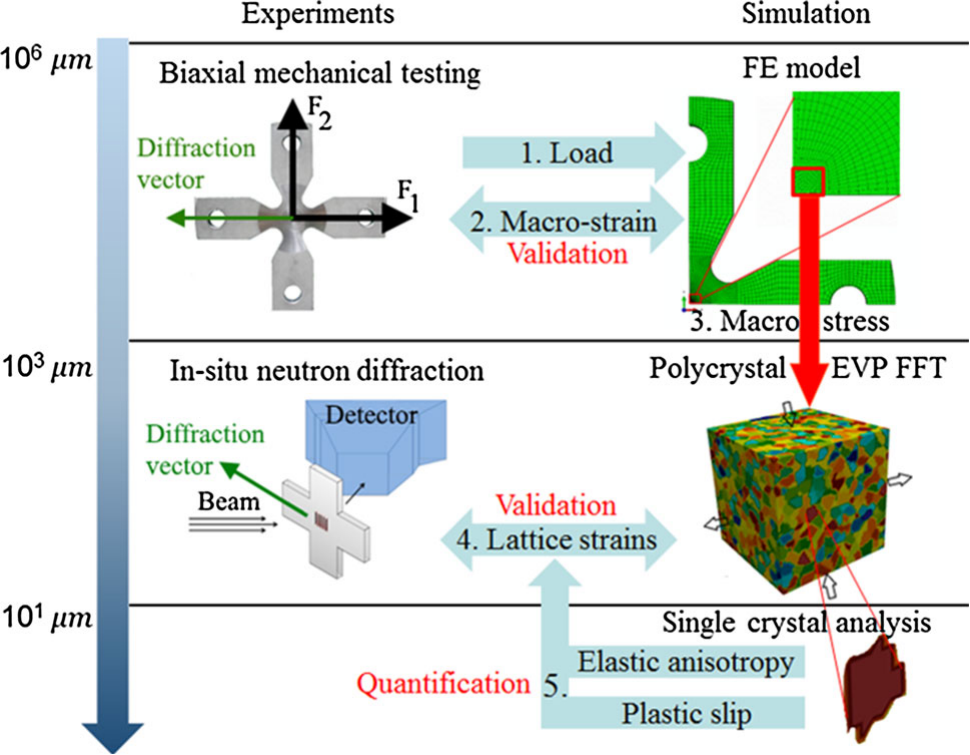
Multiscale synergy between in-situ neutron measurements during biaxial testing and FE-FFT modeling

To that end, a multiscale modeling strategy was recently proposed in Ref. 6 and is illustrated in Fig. 1. The approach involves supplying an experimental applied load and displacement conditions as boundary conditions to drive a macroscale FE simulation of cruciform geometry using the ABAQUS software. The predicted gauge surface strains are validated by using digital image correlation (DIC) measurements. The predicted macroscopic gauge stresses are supplied as homogeneous boundary conditions to drive the EVP-FFT model. Then, lattice strains calculated with EVP-FFT are averaged over all the grains belonging to a grain family and compared with in-situ neutron diffraction measurements. The combined FE and EVP-FFT approach (FE-FFT) was used to study the role of uniaxial and biaxial loading on the contribution of elastic/plastic anisotropy to the average lattice strain evolution of different grain families for 316L stainless steel cruciform samples.^6^

In this work, the objective is to highlight the role of grain neighborhood interactions on the lattice strain evolution of 200 and 220 grain families during DB and EQUI loadings. The 200 and 220 families demonstrate elastically the most compliant and intermediate compliant average lattice strain response, respectively, for both loadings.^5^ Furthermore, during DB and EQUI loadings, the average lattice strain evolution in these families shows the most interesting similarities and differences.^6^ The article is divided into sections as follows. The experimental and simulation setup are first recalled. Then, the simulation procedure is validated by comparing the predicted average lattice strain evolution for the two grain families with in-situ neutron diffraction results. Next, the simulation results are used to study the lattice strain distribution within the two grain families for DB and EQUI loadings. The comparison shows that EQUI loading results in a much larger spread in lattice strain evolution in comparison with DB loading for both grain families. To appreciate the role of grain neighborhood interactions, the lattice strain evolution is studied within the subsets of the 200 and 220 grain families; the classification into subsets is based on the crystallographic orientation of the 200 and 220 grains with respect to the diffraction vector and loading directions. The results show that the contribution of the grain neighborhood to the lattice strain evolution is highly dependent on the loading conditions.

## Experiment and Simulation Setup

In the following, the material properties, experimental details, and simulation setup are briefly recalled. For details, the readers are referred to Refs. 5 and 6.

### Material Properties and Sample

**Fig. 2:**
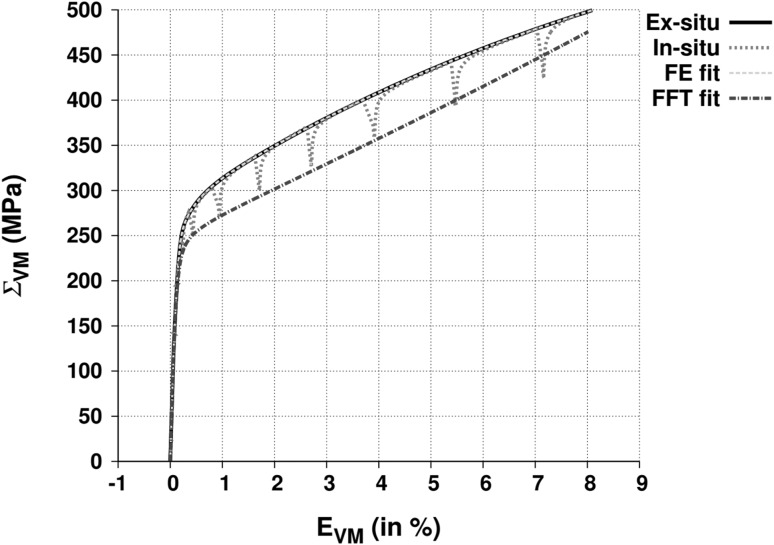
VM stress versus strain curve from uniaxial tensile loading on 316L stainless steel dog-bone samples. In dots, the experimental curve is obtained during in-situ neutron diffraction measurements. In solid black, the experimental curve is obtained during ex-situ monotonic loading. The dashed gray line (overlapping the black line) is the macroscopic FE simulation fit, and the dot-dash line is the EVP-FFT fit

The material is a warm-rolled, face-centered cubic (fcc) 316L stainless steel composed of Cr-17.25, Ni-12.81, Mo-2.73, Mn-0.86, Si-0.53, C-0.02 wt.%. Electron backscattering diffraction reveals a mild texture, the details of which are presented in Ref. 5. The grains are equiaxed with ~7 microns average size. The von Mises (VM) true stress versus strain curve from a DB tensile test is shown in solid black in Fig. 2. The cruciform geometry is shown in Fig. 1. Direction 1 is aligned along the rolling direction for both cruciform and DB samples. The cruciform sample has a central gauge thickness of 3 mm and an arm thickness of 10 mm. A two-camera system is used to perform in-situ DIC surface strain measurements. The DIC speckle pattern is designed by hand spraying layers of black and white spray paint. A homogeneous pattern is obtained with the following order of spraying: white–black–white. The spatial resolution for strain measurements is 150 × 150 *μ*m^2^. The error in DIC strain measurement scales according to the equation: ${\text{err}}\left( \% \right) = a \times E\left( \% \right) + b\left( \% \right)$, where *E* is the true strain along one of the in-plane directions and *a* and *b* fall in the range [0.014, 0.024] and [0.05, 0.09], respectively. In the present work, EQUI loading, i.e., *F*_2_:*F*_1_ = 1:1, of cruciform samples is compared with uniaxial tensile DB loading. Both tests are performed under load control at a rate of 40 N s^−1^.

### In-Situ Neutron Diffraction

Neutron diffraction experiments were performed at the pulse overlap time-of-flight diffractometer (POLDI) beamline in the Swiss spallation neutron source (SINQ) facility. The incoming beam, having a cross section of 3.8 × 3.8 mm^2^, is incident at the center of the circular gauge area. The detector and samples are oriented such that the diffraction vector $\vec{\varvec{g}}$ lies along direction 1 of cruciform sample and along the DB loading direction. An *hkl* diffraction peak is obtained when the normal $\vec{\varvec{n}}$ to the {*hkl*} planes is closely aligned with $\vec{\varvec{g}}$. The peak position of each *hkl* reflection determines the average interplanar spacing *d*_*hkl*_ for an *hkl* grain family with $\vec{\varvec{n}}$ parallel to $\vec{\varvec{g}}$. Average lattice strain for this *hkl* grain family is then determined as:

1 $$ \varepsilon_{hkl} = \frac{{d_{hkl} - d_{hkl}^{0} }}{{d_{hkl}^{0} }} $$

where $d_{hkl}^{0}$ is the initial average interplanar spacing of the *hkl* family. The detector only measures those grains that have their normal to the {*hkl*} plane oriented at ±7.5° with respect to direction 1.

During neutron measurements, sample arms are held under constant displacement resulting in stress relaxations. The dotted curve in Fig. 2 shows VM stress versus strain curve obtained from a DB test during in-situ neutron diffraction.

### Macroscale FE Simulations

ABAQUS FE simulations^20^ are used to obtain gauge stresses. Only one eighth of DB and cruciform geometries are simulated. A linear 8-node hexahedron mesh is used. Macroscopic elastic properties of 316L steel obtained from monotonic DB tests are assigned to the simulated geometry. The macroscopic elastic response is modeled as isotropic with the experimentally measured Young’s modulus of 190 GPa and Poisson’s ratio of 0.31. The plastic response is modeled with built-in nonlinear isotropic and kinematic hardening law with five back-stresses. Stress versus strain curve from monotonic tensile test (black line in Fig. 1) on DB samples is provided as input. The ABAQUS/Standard algorithm uses this curve to fit the back-stress parameters without the need for manual fitting. The FE fit is shown with a gray dashed line in Fig. 2.

### Mesoscale EVP-FFT Model

The EVP-FFT approach^19^ models the periodic representative volume element (RVE) of the polycrystalline domain. The RVE is divided into evenly spaced voxels along the sample reference directions such that each grain contains several voxels. Single-crystal elastic and plastic properties are attributed to each voxel. The elastic behavior is modeled with Hooke’s law $\varvec{\sigma}= \varvec{c}:\left( {\varvec{\varepsilon}-\varvec{\varepsilon}^{\varvec{p}} } \right),$ and the viscoplastic behavior is modeled with a power law relationship:^19^

2 $$ \dot{\varvec{\varepsilon }}^{\varvec{p}} = \mathop \sum \limits_{\text{s}} \dot{\gamma }_{0} \left( {\frac{{\left| {\varvec{m}^{\varvec{s}} :\varvec{\sigma}} \right|}}{{\tau_{\text{c}}^{\text{s}} }}} \right)^{n} {\text{sgn}}\left( {\varvec{m}^{\varvec{s}} :\varvec{\sigma}} \right) $$

where $\varvec{\sigma},  \varvec{c},  \dot{\varvec{\varepsilon }}^{\varvec{p}}$, and $\varvec{m}^{\varvec{s}}$ are local stress, elastic stiffness, viscoplastic strain rate, and Schmid tensor for slip system *s*, respectively. $\dot{\gamma }_{0}$, *n*, and $\tau_{\text{c}}^{\text{s}}$ are the reference shear rate, power law exponent, and critical resolved shear stress (CRSS) for slip system *s*, respectively. The evolution of CRSS is modeled as a function of the total accumulated shear (Γ) on all slip systems with the extended Voce-type hardening law:^21^

3 $$ \tau_{\text{c}}^{\text{s}} = \tau_{0}^{\text{s}} + \left( {\tau_{1}^{\text{s}} + \theta_{1} {\varGamma }} \right)\left( {1 - \exp \left( { - \left| {\frac{{\theta_{0}^{\text{s}} }}{{\tau_{1}^{\text{s}} }}} \right|{\varGamma}} \right)} \right) $$

where $\tau_{0}^{\text{s}} ,\left( {\tau_{0}^{\text{s}} + \tau_{1}^{\text{s}} } \right),\theta_{0}^{\text{s}}$, and $\theta_{1}^{\text{s}}$ are the initial CRSS, the back extrapolated stress, and the initial and final hardening slopes for a given slip system *s*, respectively. A detailed explanation of the FFT numerical approach is given in Refs. 19 and 22.

A 2,500-grain Voronoi tessellated microstructure with random texture is divided into 64^3^ voxels. Each voxel is assigned single-crystal properties of face-centered-cubic 316L steel.^6^ The three independent elastic constants for this steel are *c*_11_ = 204.6 GPa, *c*_12_ = 137.7 GPa, and *c*_44_ = 126.2 GPa. The hardening parameters are fit to obtain an artificial stress–strain curve that overlaps with the cusps during in-situ neutron measurements (dot-dash line in Fig. 2); this is typically done in crystal plasticity modeling of in-situ diffraction tests.^23^ The fitted extended Voce hardening law parameters are shown in Table I. Macroscopic stress boundary conditions obtained from FE simulations are used to drive the EVP-FFT model.

**Table I:** Fitted Voce hardening parameters for 316L stainless steel

$\varvec{\tau}_{0}^{\varvec{s}}$ (MPa)	$\varvec{\tau}_{1}^{\varvec{s}}$ (MPa)	$\varvec{\theta}_{0}^{\varvec{s}}$ (MPa)	$\varvec{\theta}_{1}^{\varvec{s}}$ (MPa)
50	70	105,000	410

### Virtual Diffraction

In the polycrystalline reference frame, $\vec{\varvec{g}}$ is aligned along the loading direction for the DB sample and the loading direction 1 for the cruciform sample. Note that the EVP-FFT model provides detailed information on the lattice strain or microstrain (*με*) evolution including for those grains that are out of neutron detector range. Nevertheless, for comparison with in-situ neutron diffraction, only those voxels are chosen that have one of their {*hkl*} planes nearly aligned (±7.5° tolerance) with $\vec{\varvec{g}}$. In the simulation (experiment), during plastic deformation, some voxels (grains or parts of grains) in the simulated (experimental) microstructure will move in or out of the detector angular range as a result of plastic slip-induced rotations. To facilitate a comparison with in-situ neutron diffraction results, the number of voxels contributing to the lattice strain evolution is updated after every time step. Following this comparison in the section titled “Average Lattice Strain Evolution in 200 and 220 Families,” only those voxels are considered that contributed to the lattice strain evolution prior to deformation. This is explained in detail in the section titled “Lattice Strain Distribution in 200 and 220 Families.” For all such voxels, *με* is computed as $\vec{\varvec{g}} \cdot\varvec{\varepsilon}^{\varvec{e}} \cdot \vec{\varvec{g}}$. Then $\left\langle {\left\langle {\mu \varepsilon } \right\rangle } \right\rangle_{hkl}$—here the inner brackets indicate averaging over all voxels from one grain belonging to the *hkl* family, and the outer brackets indicate averaging over all grains belonging to the same *hkl* family—should correspond to the experimentally measured *ε*_*hkl*_ from Eq. 1. Finally, in the present work, *με* is defined as 10^6^ times the lattice strain; the lattice strains are of the order 10^−3^; therefore, any quantity computed from *με* will be of the order 10^3^. This is typically done to compare with experimental lattice strains that are often presented as *ε*_*hkl*_ × 10^6^.

## Results

The FE-FFT model has already been successfully validated for DB and EQUI loading by comparing at the macroscale the FE predicted and experimental DIC strains.^6^ To avoid redundancy, the macroscale validation is not repeated. Henceforth, macroscale and mesoscale quantities are denoted with uppercase and lowercase letters, respectively.

### Macroscopic Stresses and Strains

**Fig. 3:**
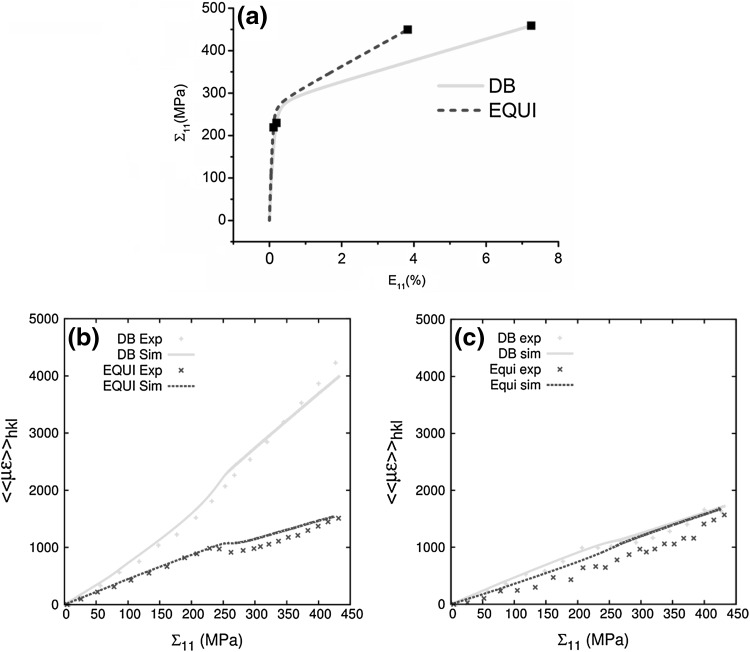
(a) FE predicted Σ_11_ versus *E*_11_ plot for DB and EQUI loading. FFT predicted $\left\langle\left\langle {\mu \varepsilon } \right\rangle\right\rangle_{hkl}$ versus FE predicted Σ_11_ in (b) 200 and (c) 220 families during DB and EQUI loadings compared with in-situ neutron diffraction experiments

Figure 3a shows the FE predicted stress versus strain along loading direction 1, i.e., Σ_11_ versus *E*_11_ curves for DB and EQUI loadings. EQUI loading results in a stiffer elastic and a harder plastic response in comparison with DB loading. This is because in the elastic regime, as a result of Poisson’s (ratio *v*) compression, $E_{11}^{\text{EQUI}} = E_{11}^{\text{DB}} \left( {1 - v} \right)$ for the same Σ_11_. In the plastic regime, for the same VM stress and equivalent plastic strain, $E_{11}^{{p,{\text{EQUI}}}} = E_{11}^{{p,{\text{DB}}}} /2$; the superscript *p* denotes the plastic component.

### Average Lattice Strain Evolution in 200 and 220 Families

Figures 3b and c show, for the 200 and 220 families, the comparison between simulation predicted $\left\langle {\left\langle {\mu \varepsilon } \right\rangle } \right\rangle_{hkl}$ and experimental *ε*_*hkl*_ as a function of FE predicted macroscopic stress Σ_11_ under both loadings. In general, a good agreement is obtained between simulations and experiments. The noisy behavior of 220 lattice strains is a result of the decrease in 220 peak intensity inducing uncertainties during the fitting.^6^ In the elastic regime, EQUI loading results in a stiffer response for both families. For both loadings, the 220 response is stiffer than the 200 response. The difference in $\left\langle {\left\langle {\mu \varepsilon } \right\rangle } \right\rangle_{hkl}$ between both loadings is most pronounced for the 200 family, which suggests a significant role of the elastic anisotropy and grain interactions. During the elastic–plastic transition between Σ_11_ = 200 and 300 MPa, the $\left\langle {\left\langle {\mu \varepsilon } \right\rangle } \right\rangle_{hkl}$ evolution under EQUI loading deviates toward that of DB loading for the 220 family. This is not the case for the 200 family, where the difference between DB and EQUI increases. The different behavior is ascribed to heterogeneous load sharing between different grain families as a result of elastic–plastic anisotropy and grain interactions.^7^–^9^ Following the elastic–plastic transition, the $\left\langle {\left\langle {\mu \varepsilon } \right\rangle } \right\rangle_{hkl}$ response for both grain families under both loadings deviates toward the original elastic slope. At the end of loading, the 220 family has nearly equal $\left\langle {\left\langle {\mu \varepsilon } \right\rangle } \right\rangle_{hkl}$ for both loadings, whereas there is a large difference for the 200 family.

### Lattice Strain Distribution in 200 and 220 Families

The results in Fig. 3 indicate that the $\left\langle {\left\langle {\mu \varepsilon } \right\rangle } \right\rangle_{hkl}$ evolution under a biaxial stress ratio *R* = Σ_22_/Σ_11_ depends on the interplay among (1) *R*, (2) elastic anisotropy, (3) plastic slip activity on each slip system, and (4) elastic/plastic grain neighborhood interactions. The role of *R* on the contribution of elastic anisotropy and plastic slip has been extensively studied in Ref. 6. In the following, the role of *R* on the contribution of grain neighborhood interactions to lattice strain evolution is highlighted. Understanding this requires going beyond the spatial resolution achievable from in-situ neutron diffraction experiments. Therefore, for the remainder of this article, the analysis will be performed solely by using the simulation results. This study complements the work done in Ref. 6.

In an in-situ neutron diffraction experiment, some parts of, or entire grains that contributed to, $\left\langle {\left\langle {\mu \varepsilon } \right\rangle } \right\rangle_{hkl}$ in the elastic regime may move out of the neutron detector range in the plastic regime because of plastic slip-induced lattice rotation. On the other hand, new grains may move within the detector range. The simulated $\left\langle {\left\langle {\mu \varepsilon } \right\rangle } \right\rangle_{hkl}$ evolution shown in Fig. 3 accounts for this evolution. Nevertheless, for a clearer understanding, in the following, we focus only on the set of *hkl* grains that in the elastic regime contributed to the simulated $\left\langle {\left\langle {\mu \varepsilon } \right\rangle } \right\rangle_{hkl}$. The analysis is performed on the same voxels (grains) in both elastic and plastic regimes under the two loadings. Note that in the plastic regime, some of these voxels (grains) may move out of the detector angular range. In doing this, we eliminate possible influences of the detector geometries that are inherent in the experimental setup.

**Fig. 4:**
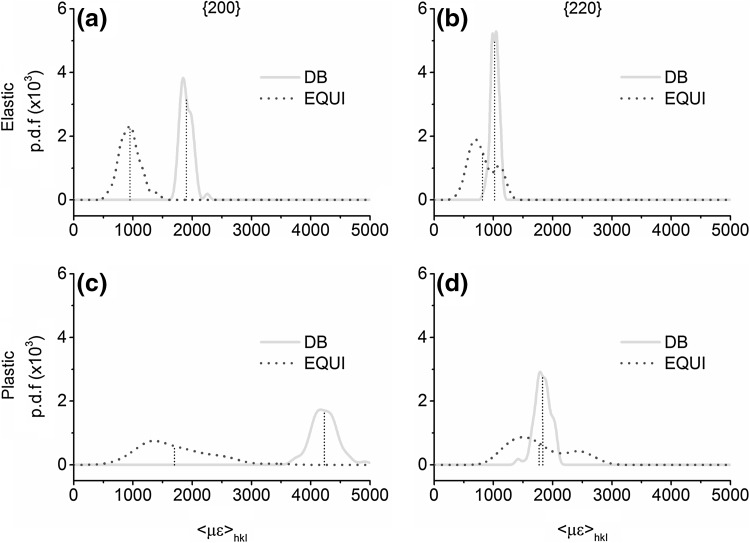
p.d.f. of $\left\langle {\mu \varepsilon } \right\rangle_{hkl}$ in (a, c) 200 and (b, d) 220 grain families in the (a, b) elastic and (c, d) plastic regimes under DB and EQUI loadings

As a first step to understand the role of *R* on grain neighborhood interactions, the $\left\langle {\mu \varepsilon } \right\rangle_{hkl}$ distribution within 200 and 220 families is studied; $\left\langle { } \right\rangle_{hkl}$ represents averaging over all voxels of only one grain belonging to an *hkl* family. Note that $\left\langle {\mu \varepsilon } \right\rangle_{hkl}$ smears out intragranular distributions of lattice strains. Its value depends on grain neighborhood interactions. Figure 4 shows the probability distribution function (p.d.f.) of $\left\langle {\mu \varepsilon } \right\rangle_{200}$ and $\left\langle {\mu \varepsilon } \right\rangle_{220}$ for all grains contributing to the 200 and 220 families, respectively, under both loadings in both regimes.

All p.d.f. peaks are asymmetric with a pronounced right shoulder. In some cases, there are distinguishable second peaks. This implies that subsets of 200 and 220 grains experience different stress states and, consequently, different $\left\langle {\mu \varepsilon } \right\rangle_{hkl}$. When going from the elastic to the plastic regime, the width of the $\left\langle {\mu \varepsilon } \right\rangle_{hkl}$ distribution increases in both families and for both loadings. After plastic deformation, the difference in mean $\left\langle {\mu \varepsilon } \right\rangle_{200}$ and mean $\left\langle {\mu \varepsilon } \right\rangle_{220}$ between the two loadings increases and decreases, respectively. Consequently, under the two loadings, there is negligible overlap in p.d.f.($\left\langle {\mu \varepsilon } \right\rangle_{200}$) but considerable overlap in p.d.f.($\left\langle {\mu \varepsilon } \right\rangle_{220}$). EQUI loading results in a wider $\left\langle {\mu \varepsilon } \right\rangle_{hkl}$ spread among the grains for both grain families. To understand this better, in the next two sections, a classification scheme is presented to subdivide the *hkl* grain families according to their crystallographic orientation with respect to the diffraction vector. Then, a graphical analysis of the $\left\langle {\mu \varepsilon } \right\rangle_{hkl}$ spread between grains belonging to these subsets is performed.

### Classification of *hkl* Families Based on Crystallographic Orientation

**Fig. 5:**
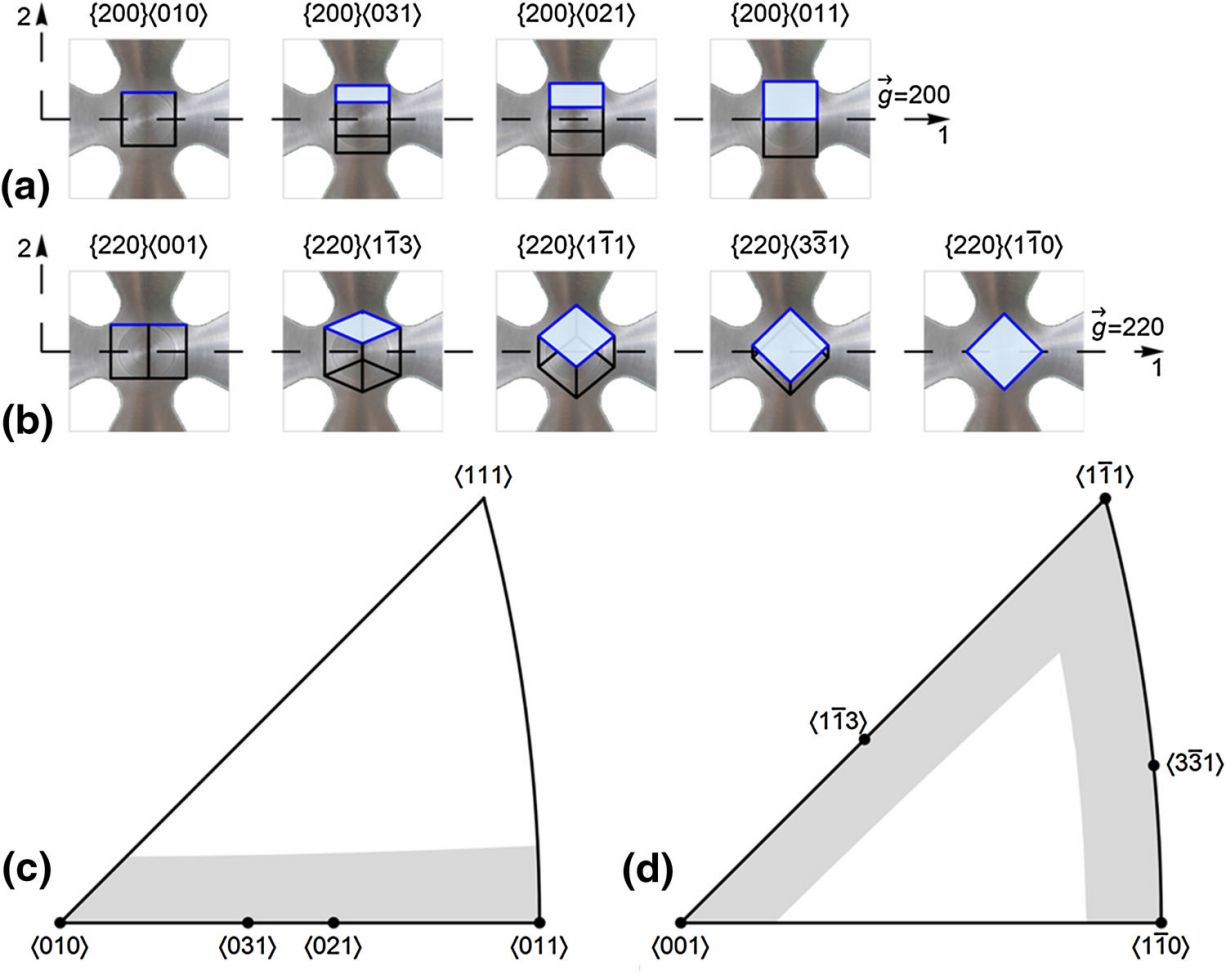
(a) $\left\{ {200} \right\}\left\langle {a_{i} b_{i} c_{i} } \right\rangle$ and (b) $\left\{ {220} \right\}\left\langle {a_{i} b_{i} c_{i} } \right\rangle$ subset unit-cell orientations with respect to cruciform in-plane loading directions and $\vec{g}$. The shaded surface facilitates the visualization of the unit-cell rotation about $\vec{g}$ for different subsets. 〈100〉 symmetric stereographic triangles showing the shaded region containing $\left\{ {hkl} \right\}\left\langle {a_{i} b_{i} c_{i} } \right\rangle$ subsets of the (c) 200 and (d) 220 families at ±7.5° with respect to direction 1

An *hkl* grain family has the loading direction 1 and $\vec{\varvec{g}}$ normal to an {*hkl*} plane. Every *hkl* family can be subdivided based on crystallographic orientations of its constituent grains about $\vec{\varvec{g}}$. Figure 5 graphically shows the 200 and 220 grain unit cells with respect to their orientation relative to $\vec{\varvec{g}}$. These grains form part of the subset $\left\{ {200} \right\}\left\langle {a_{i} b_{i} c_{i} } \right\rangle$ and $\left\{ {220} \right\}\left\langle {a_{i} b_{i} c_{i} } \right\rangle$ of the 200 and 220 families, and they lie in the shaded regions shown on the symmetric stereographic triangles in Fig. 5.

### Lattice Strain Distribution within 200 and 220 Subsets

**Fig. 6:**
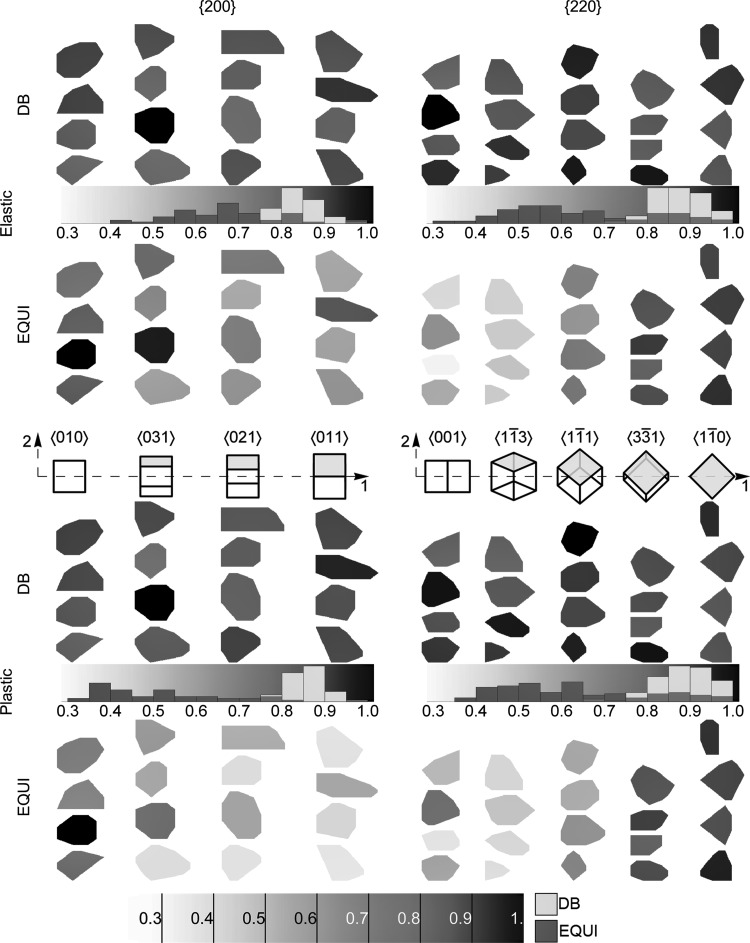
Graphic representation of $\overline{\left\langle {\mu \varepsilon } \right\rangle_{{\left\{ {hkl} \right\}\left\langle {a_{i} b_{i} c_{i} } \right\rangle }}}$ distribution within $\left\{ {200} \right\}\left\langle {a_{i} b_{i} c_{i} } \right\rangle$ and $\left\{ {220} \right\}\left\langle {a_{i} b_{i} c_{i} } \right\rangle$ subsets during DB and EQUI loadings in elastic and plastic regime. Each column contains two-dimensional projections, along the out-of-plane direction 3 in Figs. 5a and b, of some randomly selected grains belonging to the $\left\{ {hkl} \right\}\left\langle {a_{i} b_{i} c_{i} } \right\rangle$ subsets. The grains are color coded according to their $\overline{\left\langle {\mu \varepsilon } \right\rangle_{{\left\{ {hkl} \right\}\left\langle {a_{i} b_{i} c_{i} } \right\rangle }}}$ values. The scale is shown at the bottom of the figure as well as with the histograms within the figure. The histograms show the difference in $\overline{\left\langle {\mu \varepsilon } \right\rangle_{{\left\{ {hkl} \right\}\left\langle {a_{i} b_{i} c_{i} } \right\rangle }}}$ distribution for an *hkl* family in both regimes subjected to DB and EQUI loadings

To understand the distribution of $\left\langle {\mu \varepsilon } \right\rangle_{hkl}$ between subsets of 200 and 220 families, the $\left\langle {\mu \varepsilon } \right\rangle_{{\left\{ {hkl} \right\}\left\langle {a_{i} b_{i} c_{i} } \right\rangle }}$ distribution is analyzed; here $\left\langle {} \right\rangle_{ \left\{ {hkl} \right\}\left\langle a_{i} b_{i} c_{i} \right\rangle}$ implies averaging over all voxels belonging to one grain in an $\left\{ {hkl} \right\}\left\langle {a_{i} b_{i} c_{i} } \right\rangle$ subset. For brevity, the analysis is performed only on $\left\{ {200} \right\}\left\langle {010} \right\rangle ,\left\{ {200} \right\}\left\langle {031} \right\rangle ,\left\{ {200} \right\}\left\langle {021} \right\rangle ,\left\{ {200} \right\}\left\langle {011} \right\rangle ,\left\{ {220} \right\}\left\langle {001} \right\rangle ,\left\{ {220} \right\}\left\langle {1\bar{1}3} \right\rangle ,\left\{ {220} \right\}\left\langle {1\bar{1}1} \right\rangle ,\left\{ {220} \right\}\left\langle {3\bar{3}1} \right\rangle$ and $\left\{ {220} \right\}\left\langle {1\bar{1}0} \right\rangle$ subsets. Figure 6 shows the graphical distribution of $\overline{{\left\langle {\mu \varepsilon } \right\rangle_{{\left\{ {hkl} \right\}\left\langle {a_{i} b_{i} c_{i} } \right\rangle }} }}$ within four different grains belonging to each subset in both regimes for both loadings. Here the $\overline{{\left\langle {\mu \varepsilon } \right\rangle_{{\left\{ {hkl} \right\}\left\langle {a_{i} b_{i} c_{i} } \right\rangle }} }}$ represents $\left\langle {\mu \varepsilon } \right\rangle_{{\left\{ {hkl} \right\}\left\langle {a_{i} b_{i} c_{i} } \right\rangle }}$ normalized by using $\hbox{max} \left( {\left\langle {\mu \varepsilon } \right\rangle_{{\left\{ {hkl} \right\}}} } \right)$ from a single *hkl* family in one regime under a given load. For example, $\overline{{\left\langle {\mu \varepsilon } \right\rangle_{{\left\{ {200} \right\}\left\langle {010} \right\rangle }} }}$ for DB loading in the elastic regime is normalized using $\hbox{max} \left( {\left\langle {\mu \varepsilon } \right\rangle_{{\left\{ {200} \right\}}} } \right)$ for DB loading in the elastic regime. Note that the grain with $\hbox{max} \left( {\left\langle {\mu \varepsilon } \right\rangle_{{\left\{ {hkl} \right\}}} } \right)$ may not appear in Fig. 6.

The following arguments can be made on the mean $\overline{{\left\langle {\mu \varepsilon } \right\rangle_{{\left\{ {hkl} \right\}\left\langle {a_{i} b_{i} c_{i} } \right\rangle }} }}$ response of each $\left\{ {hkl} \right\}\left\langle {a_{i} b_{i} c_{i} } \right\rangle$ subset of an *hkl* family. Under EQUI loading in both regimes, the following descending order for mean $\overline{{\left\langle {\mu \varepsilon } \right\rangle_{{\left\{ {200} \right\}\left\langle {a_{i} b_{i} c_{i} } \right\rangle }} }}$ within the 200 family is observed: $\left\{ {200} \right\}\left\langle {010} \right\rangle$, $\left\{ {200} \right\}\left\langle {031} \right\rangle$, $\left\{ {200} \right\}\left\langle {021} \right\rangle ,$ and $\left\{ {200} \right\}\left\langle {011} \right\rangle$. The ordering for mean $\overline{{\left\langle {\mu \varepsilon } \right\rangle_{{\left\{ {220} \right\}\left\langle {a_{i} b_{i} c_{i} } \right\rangle }} }}$ within the 220 family is $\left\{ {220} \right\}\left\langle {1\bar{1}3} \right\rangle$, $\left\{ {220} \right\}\left\langle {001} \right\rangle$, $\left\{ {220} \right\}\left\langle {1\bar{1}1} \right\rangle$, $\left\{ {220} \right\}\left\langle {1\bar{1}0} \right\rangle$, and $\left\{ {220} \right\}\left\langle {3\bar{3}1} \right\rangle$. Under DB loading, for both families, there is no clear ordering scheme in the elastic or plastic regime.

The grain colors in Fig. 6 show that the width of $\overline{{\left\langle {\mu \varepsilon } \right\rangle_{{\left\{ {hkl} \right\}\left\langle {a_{i} b_{i} c_{i} } \right\rangle }} }}$ is narrower for DB loading than for EQUI for all subsets of the same *hkl* family; the normalized histograms in Fig. 6 also confirm this. The difference in $\overline{{\left\langle {\mu \varepsilon } \right\rangle_{{\left\{ {hkl} \right\}\left\langle {a_{i} b_{i} c_{i} } \right\rangle }} }}$ distributions arises as a result of the alignment of loading directions with respect to $\vec{\varvec{g}}$. DB samples are only loaded along $\vec{\varvec{g}}$; therefore, the lattice orientation about $\vec{\varvec{g}}$ does not influence the contribution of elastic–plastic anisotropy to $\overline{{\left\langle {\mu \varepsilon } \right\rangle_{{\left\{ {hkl} \right\}\left\langle {a_{i} b_{i} c_{i} } \right\rangle }} }}$ for any $\left\{ {hkl} \right\}\left\langle {a_{i} b_{i} c_{i} } \right\rangle$ subset.^6^^,^ 7 Nevertheless, EQUI loading has a macroscopic stress component in the direction normal to $\vec{\varvec{g}}$. Then the contribution of elastic/plastic anisotropy of 316L steel and crystallographic orientation of $\left\{ {hkl} \right\}\left\langle {a_{i} b_{i} c_{i} } \right\rangle$ subsets about $\vec{\varvec{g}}$ has an influence on $\overline{{\left\langle {\mu \varepsilon } \right\rangle_{{\left\{ {hkl} \right\}\left\langle {a_{i} b_{i} c_{i} } \right\rangle }} }}$ for EQUI (*R* = 1) in comparison with DB (*R* = 0).^6^

For EQUI loading, the $\overline{{\left\langle {\mu \varepsilon } \right\rangle_{{\left\{ {hkl} \right\}\left\langle {a_{i} b_{i} c_{i} } \right\rangle }} }}$ spread within the same $\left\{ {hkl} \right\}\left\langle {a_{i} b_{i} c_{i} } \right\rangle$ subset increases going from the elastic to the plastic regime. Nonetheless, such a trend is not clear for DB loading. These results imply that along with elastic–plastic anisotropy, grain neighborhood interactions have an important contribution to the load-carrying capacity of different grains belonging to the same $\left\{ {hkl} \right\}\left\langle {a_{i} b_{i} c_{i} } \right\rangle$ subset, and this contribution increases from DB to EQUI loading.

## Conclusion

A multiscale FE-FFT elastic–plastic model was used to understand the differences in intergranular strain evolution of the 200 and 220 grain families during uniaxial dog-bone and equibiaxial cruciform loading of 316L stainless steel. The distribution of lattice strain averaged over a grain belonging to an *hkl* family, $\left\langle {\mu \varepsilon } \right\rangle_{hkl}$, and belonging to $hkl\left\langle {a_{i} b_{i} c_{i} } \right\rangle$ subsets, $\left\langle {\mu \varepsilon } \right\rangle_{{\left\{ {hkl} \right\}\left\langle {a_{i} b_{i} c_{i} } \right\rangle }}$, is analyzed. The following are the main results of the study:

1. The $\left\langle {\mu \varepsilon } \right\rangle_{hkl}$ distribution for both families in the elastic regime shows that equibiaxial loading results in a wider distribution of $\left\langle {\mu \varepsilon } \right\rangle_{hkl}$. The width of the $\left\langle {\mu \varepsilon } \right\rangle_{hkl}$ distribution increases for both loadings in the plastic regime. The combined effect of elastic–plastic anisotropy significantly affects the $\left\langle {\mu \varepsilon } \right\rangle_{hkl}$ distribution for both grain families and is higher for equibiaxial (biaxial stress ratio *R* = 1) loading compared with dog-bone (*R* = 0) loading. Therefore, it can be expected that these effects vary according to *R*.
2. Grain neighborhood plays an important role on $\left\langle {\mu \varepsilon } \right\rangle_{{\left\{ {hkl} \right\}\left\langle {a_{i} b_{i} c_{i} } \right\rangle }}$ distribution for grains belonging to the same $\left\{ {hkl} \right\}\left\langle {a_{i} b_{i} c_{i} } \right\rangle$ subset and is significantly higher for equibiaxial loading in comparison with dog-bone loading, and it may also depend on *R*.
